# Sensitivity of the COPD assessment test (CAT questionnaire) investigated in a population of 681 consecutive patients referring to a lung clinic: the first Italian specific study

**DOI:** 10.1186/2049-6958-9-15

**Published:** 2014-03-15

**Authors:** Roberto W Dal Negro, Luca Bonadiman, Paola Turco

**Affiliations:** 1 CESFAR - Centro Studi Nazionale di Farmacoeconomia e Farmacoepidemiologia Respiratoria, Verona, Italy; 2 Research & Clinical Governance, Verona, Italy

**Keywords:** CAT questionnaire, COPD, COPD impact, Long-term monitoring, Respiratory health

## Abstract

**Background:**

Chronic Obstructive Pulmonary Disease (COPD) is a major cause of morbidity and mortality at global level even if still underestimated. The insufficient use of specific tools for an objective definition and staging, the inadequate awareness of COPD, but also a difficult patient-to-doctor communication, can contribute to the poor management of COPD. A very simple, short and sensitive questionnaire (the “COPD Assessment Test” - CAT questionnaire) is now available for assessing the impact of COPD on the patient’s health. The present study was designed to provide such evidence using data generated throughout Italy.

**Methods:**

The Italian validated version of the CAT questionnaire was distributed to 681 consecutive COPD patients of different severity (males = 480), well matched for age, gender, smoking habit, geographical distribution, BMI, dyspnoea score and educational level. The CAT score variability was investigated vs all anagraphic, and clinical variables, and spirometric indices of lung function (regression). No Italian data are available to our knowledge on the CAT use, neither in General Medicine, nor in the specialist setting.

**Results:**

Data of this study confirmed that the CAT questionnaire is a sensitive, simple, and quick tool for assessing the respiratory status of COPD patients. The CAT score proved not conditioned by the patient’s age, gender, body size, geographical origin, and educational level. It was inversely correlated with the spirometric values, even if not uniquely linked to them.

**Conclusions:**

The CAT score does not represent a surrogate measurement of lung function: it is an instrument which focuses on different areas of respiratory health in COPD patients, thus providing an useful and objective tool for the long-term clinical and therapeutic monitoring of COPD patients in the specialist outpatient setting.

## Background

Chronic Obstructive Pulmonary Disease (COPD) is a major cause of morbidity and mortality at global level [1,2], and in Italy affects approximately 10% of adults over the age of 40.

The main aim of treatment for COPD is to reduce the symptoms of the disease keeping the patient as healthy as possible.

However, despite the combined efforts of the major international scientific institutions (the American Thoracic Society (ATS), the European Respiratory Society (ERS), the GOLD Guidelines) in an attempt to find the most appropriate diagnosis and treatment for COPD [3,4], this disease is still often greatly underestimated both from a diagnostic and from a therapeutic point of view [5-9].

This causes a further significant increase in the economic and socio-economic impact of COPD, mainly due to direct costs (over 70% even in Italy) relative to frequent health emergencies, hospitalizations, and loss of productivity deriving from the clinically uncontrolled disease [5-8]. From a general point of view, these findings indicate that COPD is still widely underestimated from a therapeutic viewpoint, despite its profound impact on both the patient’s physical and emotional health.

There are several concomitant factors contributing to such a poor management: the inadequate awareness of COPD as an effective disease (particularly in its early stages); the insufficient use of specific diagnostic tools (even basic tests, such as spirometry) for a more objective definition and staging; and the uneven availability of lung services that our country still suffers from; the insufficient prescription of appropriate and prolonged treatments even for patients already diagnosed; shortage of time; the general practitioner’s difficulty in timely identifying these patients, and finally, the real patient’s difficulty to communicate his/her medical condition to the doctor effectively, which is often perceived (and thus referred) incorrectly [9].

One of the most critical aspects is the lack of simple, reproducible, and specific tools, capable of raising awareness among doctors and patients concerning the true value of patient’s claimed severity of symptoms, as well as of his objective limitations in respiratory health status and quality of life.

Current specific tools (questionnaires) for measuring the quality of life are quite capable of representing the criticality of COPD patients (i.e. The St. George's Respiratory Questionnaire – SGRQ), but they take so long time to be filled in properly (even in their short form) that they are not used at all in clinical practice.

**Figure 1:**
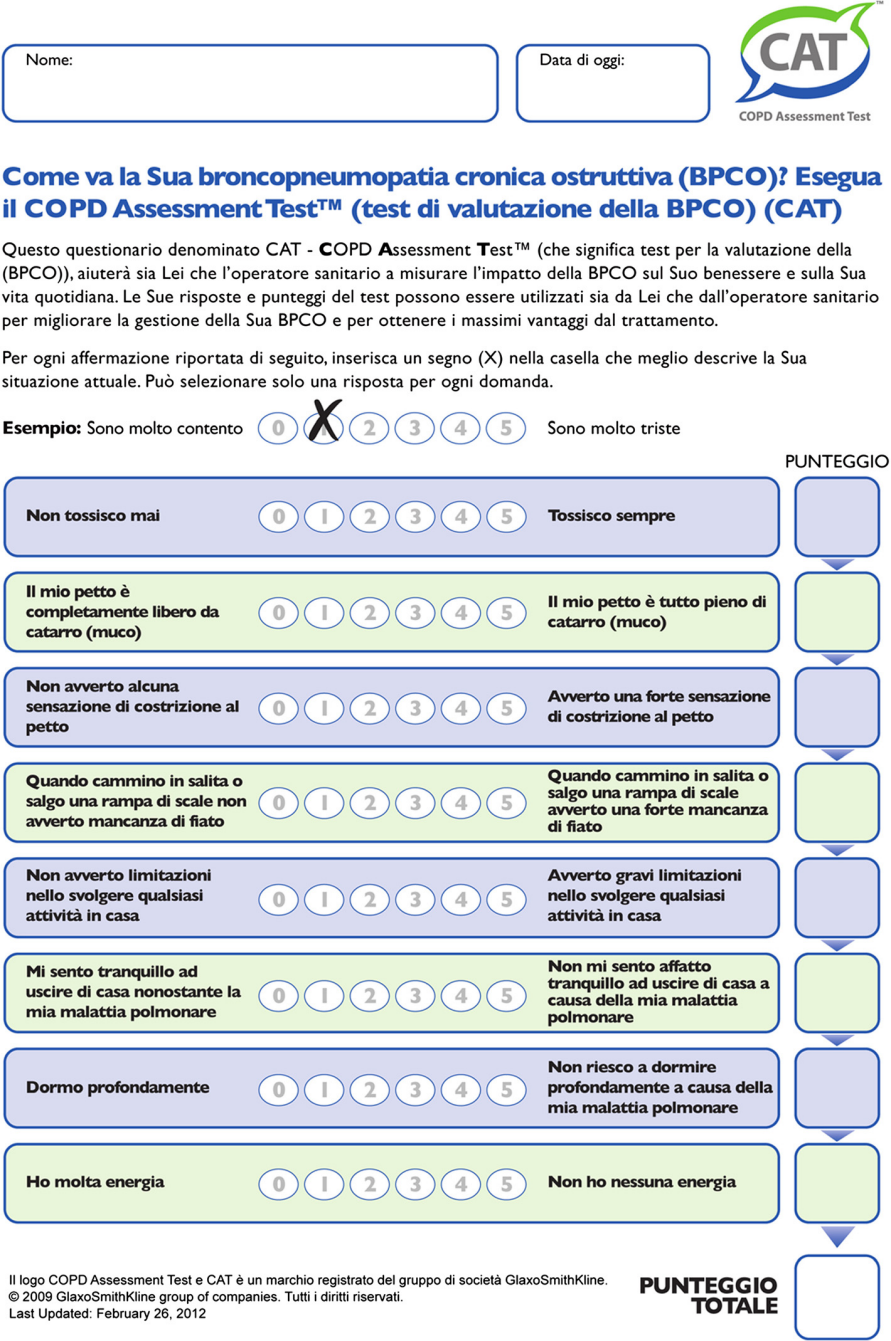
The CAT questionnaire in its validated Italian version.

Recently, a very simple, short and specific questionnaire was validated (such as the “CAT questionnaire” or COPD Assessment Test) in order to assess precisely the impact of COPD on the patient’s health. In its definitive version, it consists of eight questions (each on a scale of 0 to 5, with an overall score range of 0–40) which cover various domains of respiratory health status [10] (cough; production of secretions; tightness in the chest; shortness of breath when walking upstairs; housework; ease of living at home; quality of sleep; energy). This tool has proved to be accurate and extremely simple, easy to understand, as well as very easy and quick for the patient to fill in [10-13] (Figure 1).

As to our knowledge no Italian data are still available on the CAT use, neither in the General Medicine nor in the Pneumological context, the present study was designed to investigate the variability and the consistency of the CAT questionnaire in COPD outpatients referring to lung structures throughout Italy.

## Methods

The Italian validated version of the CAT questionnaire was distributed to consecutive subjects suffering from COPD of different clinical severity (GOLD Guidelines 2011). Each subject was assessed spirometrically (complete spirometry performed by means of CPFS/D – Medical Graphics Co.; Oak Grove Parkway, St. Paul, Minnesota, USA), and the following indices were collected: FEV_1_% predicted; Vital Capacity (VC)% predicted; MMEF% predicted; FEV_1_/FVC%; FEV_1_/VC%.

The Dyspnea score was calculated with the MRC scaleand BMI (Body Mass Index) was also calculated for each subject.

Smoking habit was taken into account (expressed as no. of packs per year), together with the subjects’ educational level; any comorbidity (in terms of type and number); the ongoing regular treatment (drugs in the R03 category of the NTP); the number of steroids and/or antibiotics courses followed in the last 12 months (such as a surrogate index of true acute exacerbations).

Statistics: the distribution of values for the patients’ age and their CAT score was checked; mean values ± SD were compared by t unpaired test; all possible relationships between the CAT scores and the values of all other variables were checked by linear regression. Due to the huge number of different comparisons, p < 0.01 was accepted as the minimum level of statistical significance.

## Results

A total of 681 consecutive COPD patients of different severity were enrolled, coming from different Italian regions. They proved geographically well matched: from the North n = 248, (36.4%); the Centre n = 209, (30.7%); the South and the islands: n = 224, (32.9%).

The general characteristics of the population are summarized in Table 1, also by gender.

**Table 1:** General characteristics of the population (n = 681)

	**Population**	**Men**	**Women**
n.	681	480	201
Age (mean, years)	72.3 ± 9.6 sd	72.9 ± 9.1 sd	70.8 ± 10.4 sd
Origin (%)			
North	36.4%	71.8%	28.2%
Center	30.7%	73.4%	26.8%
South + Islands	32.9%	75.9%	24.1%
Smoking			
smokers (%)	14.5%	13.1%	17.9%
no. of pack/years	29.3 ± 27.6 sd	35.4 ± 28.0 sd	15.1 ± 20.3 sd
ex-smokers (%)	63.9%	75.7%	35.8%
non-smokers (%)	21.6%	11.2%	46.3%
BMI	29.1 ± 5.0 sd	29.3 ± 4.6 sd	28.7 ± 5.9 sd

**Figure 2:**
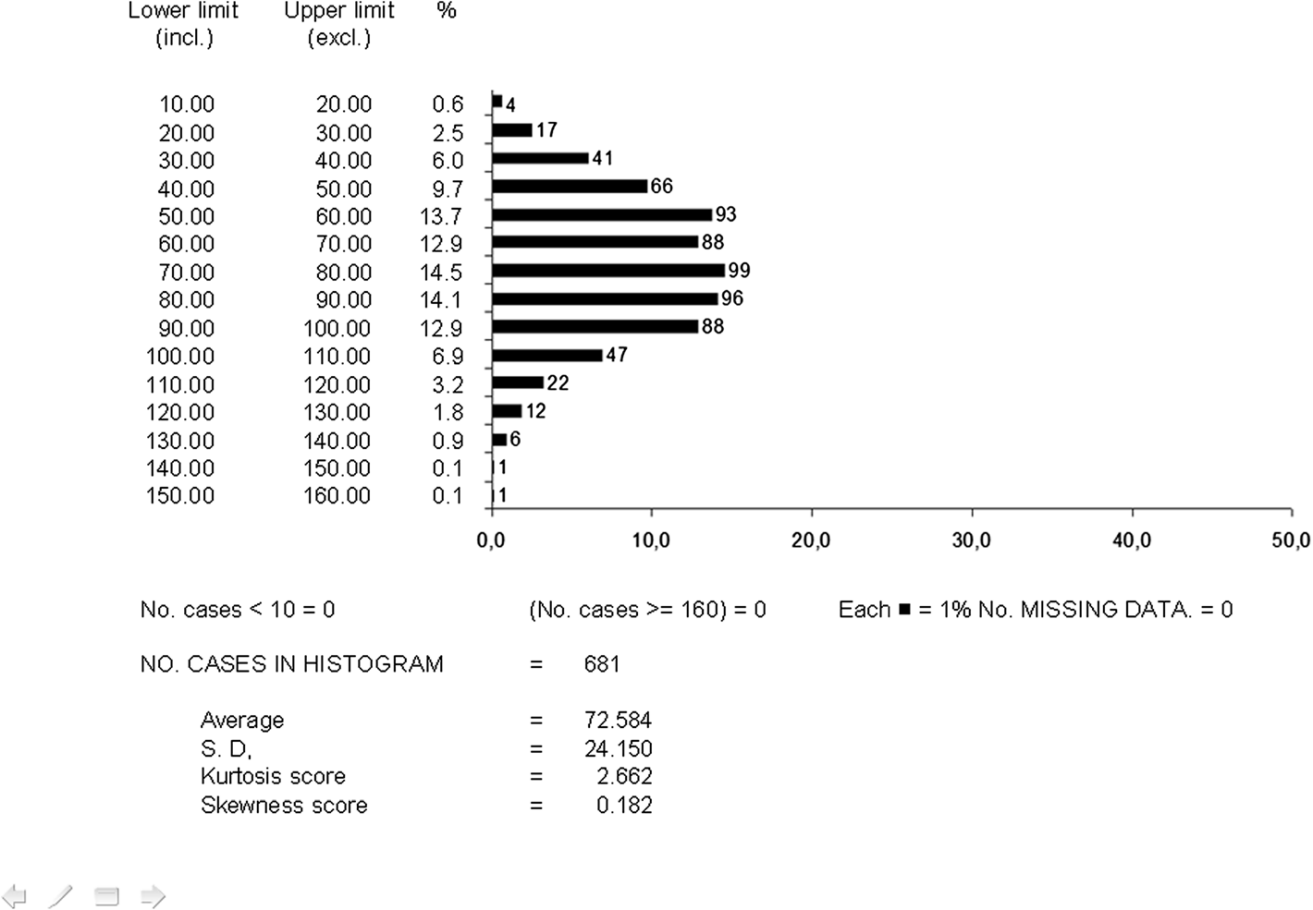
**Distribution of FEV**_**1**_**% predicted values (by 10% intervals) for the total sample (n = 681).**

Table 2 reports mean values of lung function measured in the population, together with the corresponding mean value of MRC and the CAT score: in general terms, subjects characterized by a mild-to-moderate condition were prevailing, even though a 20% of subjects were severe (Figure 2). Males appeared slightly more compromised than females, but no significant difference was seen by gender between the corresponding CAT scores (*t* test = ns). The mean CAT score for the Italian sample was also shown to be extremely close to those for the other European countries [11,12].

**Table 2:** Lung function and MRC score (n = 681)

	**Population**	**Men**	**Women**	***t *** **test**
FEV_1_ (% pred)	72.6 ± 24.1 sd	69.7 ± 23.3 sd	79.4 ± 24.7 sd	
FVC (% pred)	88.7 ± 19.4 sd	85.2 ± 18.4 sd	97.0 ± 19.0 sd	
VC (% pred)	88.6 ± 18.7 sd	86.4 ± 18.3 sd	94.1 ± 18.5 sd	
MMEF (% pred)	33.9 ± 21.8 sd	33.9 ± 21.7 sd	33.7 ± 22.0 sd	
FEV_1_/FVC (%)	62.7 ± 13.2 sd	61.3 ± 13.1 sd	66.1 ± 12.9 sd	
FEV_1_/VC (%)	60.7 ± 14.5 sd	58.9 ± 14.4 sd	65.0 ± 13.8 sd	
MRC (score)	1.2 ± 1.0 sd	1.2 ± 1.1 sd	1.3 ± 1.0 sd	
**CAT (score)**	**16.6 ± 7.1**	**16.5 ± 7.1 sd**	**16.8 ± 7.2 sd**	**ns**

**Figure 3:**
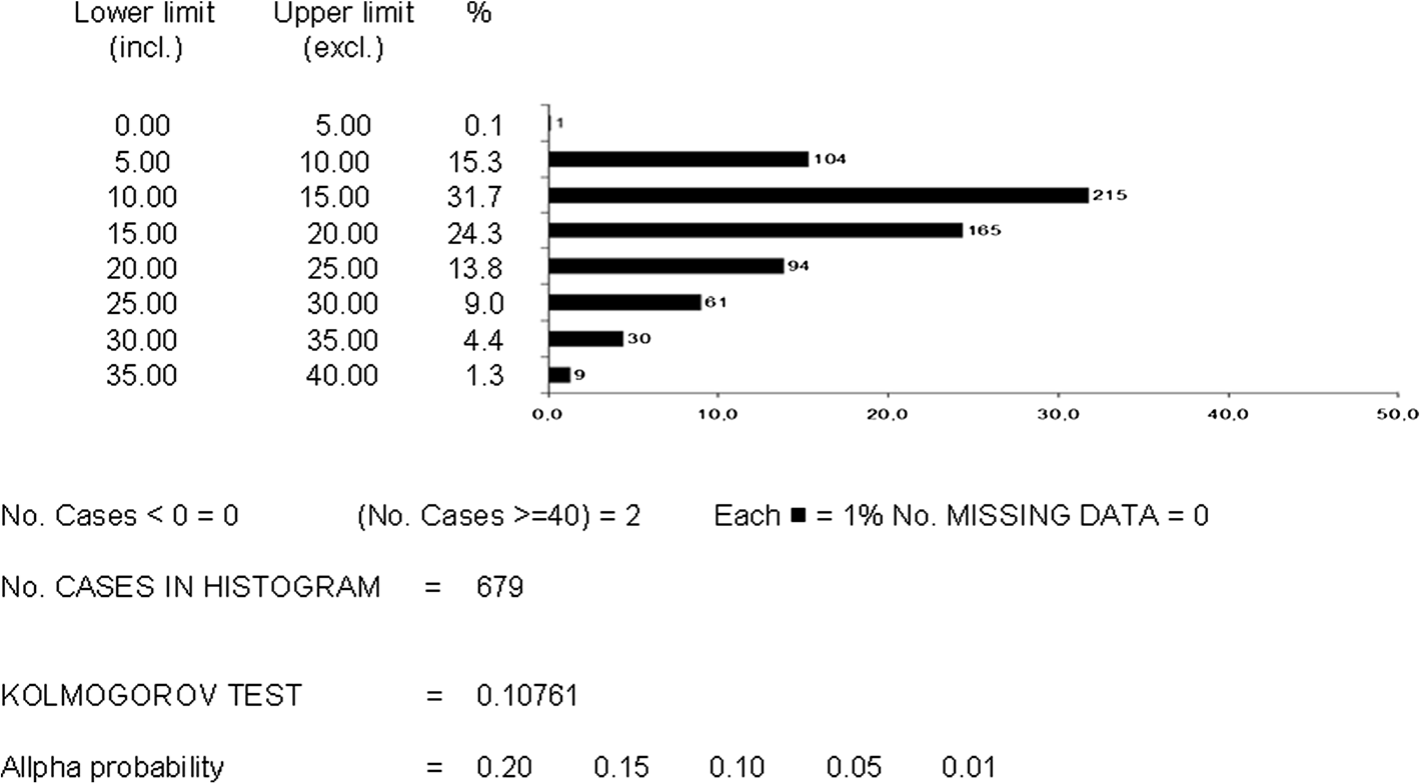
Distribution of CAT scores (by 5 point intervals) for the total sample (n = 681).

**Figure 4:**
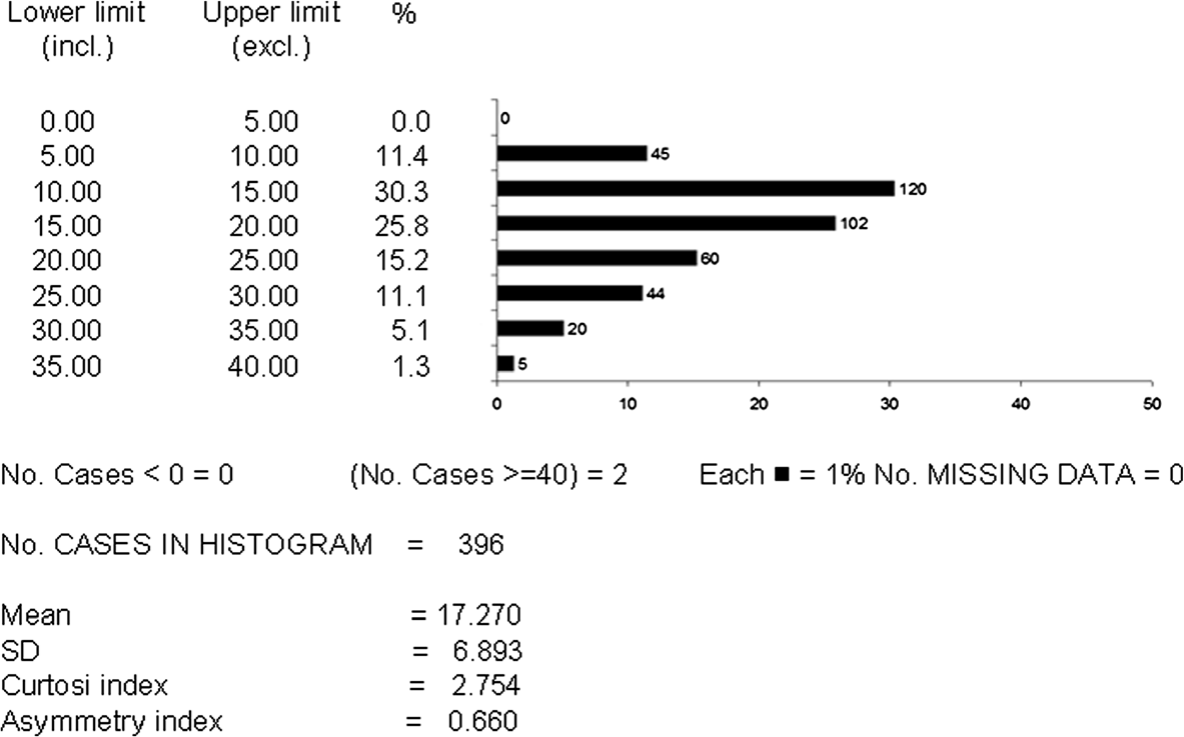
**Distribution of CAT scores (by 5 point intervals) in patients with FEV**_**1**_ **< 80% predicted.**

Figures 2 and 3 report the distribution of the FEV_1_ and CAT values respectively in the population as a whole (the corresponding mean values ± SD are those shown in Table 2). In the case of FEV_1_, it is easy to realize how the distribution of values was very close to a normal distribution, with an extremely low skewness score. The mean CAT score corresponds perfectly to that observed in other European countries [10]. The distribution of CAT values was centered in the range 5 and 25. When distribution of CAT values was limited to subjects with a FEV_1_ < 80% predicted (n = 398), the asymmetry of the distribution shape remains almost unchanged, despite a slight increase in the mean CAT score (17.3 ± 7.0 sd) (Figure 4).

The regressions calculated for the CAT score and the values of all the other variables led to the following results:

– No significant correlation was assessed between the CAT score and gender, age, BMI, and the educational level of subjects, respectively:

• vs gender: (Y = 16.2 + .26 X) p = 0.67

• vs age: (Y = 13.6 + 4.1 X) p = 0.15

• vs BMI: (Y = 13.7 + 9.8X) p = 0.07

• Vs educational level: (Y = 16.9 – 0.1X) p = 0.65

– The smoking habit proved not significant *per se*, even if a positive correlation was found for the no. pack/years, being the correlation index rather low (r = 0.08):

• vs smoking: (Y = 17.6-0.4X) p = 0.25

• vs pack/years: (Y = 15.9 + 2.1 X) p = 0.03

– A significant negative correlation was seen with all spirometric indices considered, even if the correlation indices were singularly very low:

• vs FEV_1_% (Y = 21.0 -.06X) **p < 0.001** r = -0.21

• vs VC% (Y = 23.2-7.5 X) **p < 0.001** r = -0.20

• vs MMEF% (Y = 17.1-3.3X) p = 0.008 r = -0.10

• **vs FEV**_**1**_**/FVC% (Y = 21.3-7.6X) p < 0.001** r = -0.14

• vs FEV_1_/VC% (Y = 20.4-6.4X) **p = 0.001** r = -0.13

– A strong and significant positive correlation was seen with the dyspnea score:

• vs MRC score (Y = 11.4 + 4.2 X) **p < 0.001 r = 0.62**

A confirmation for these findings comes from the CAT score, which was significantly and positively related to the extent of dyspnea at rest (p < 0.001; r = 0.27) and during exercise (p < 0.001; r = 0.37), and of wheezing (p < 0.006; r = 0.11).

– A significant positive correlation was also found with the no. of courses of systemic steroids and of antibiotics (both expression of the occurrence of acute exacerbations) in the previous 12 months, though singularly with a low correlation index:

• vs no. of steroid cycles (Y = 16.0 + 2.6 X) **p < 0.001 r = 0.19**

• vs no. of antibiotic cycles (Y = 15.9 + 1.7 X) **p < 0.001 r = 0.16**

– The infectious respiratory comorbidities (bronchial exacerbations and/or pneumonia) showed a mild positive correlation with the CAT score, even though the correlation index was quite low:

• vs no. infectious respiratory comorbidities (Y = 16.1 + 1.5 X) p = 0.02 r = 0.10

Only comorbidities of metabolic and neurological cause (but not the cardiovascular, gastroenterological, metabolic, neurological, neoplasticones) showed some correlation with the CAT score, though with very low correlation indices:

• vs no. metabolic comorbidities (Y = 16.0 + 2.0 X) **p = 0.001 r = 0.12**

• vs no. neurological comorbidities (Y = 16.4 + 2.6 X) p = 0.02 r = 0.09

A slight correlation was also found between the CAT score and the no. of comorbidities documented:

• vs no. of comorbidities (Y = 14.2 + 0.7 X) **p < 0.001 r = 0.21**

It is interesting to note that**,** when the variation of the CAT score was checked vs the current therapeutic treatment, the CAT scores proved significantly higher in the untreated patients, as well as in those treated only with short-acting β2-adrenergics on demand (p > 0.002), and in those only assuming oral xanthines (p < 0.047). No significant correlation was found in subjects undergoing regular treatment with long-term appropriate strategies, such as long-acting bronchodilators and/or inhaled corticosteroids (all p = ns).

Finally, even though the link was rather weak (r = 0.08), the variations in the CAT score proved significantly related (p < 0.030) to the no. of respiratory drugs (belonging to the R03category in the NTP) used by the patients, and thus with the complexity of their ongoing treatment.

## Discussion

Data from the present study, which was carried out in a representative sample of italian COPD outpatients referring to a lung Unit, confirm the sensitivity and the consistency of the CAT questionnaire, and lead to suggest and support its use as a quick and specific clinical tool for assessing the respiratory health status of COPD patients. The CAT questionnaire also proved to be very sensitive in capturing the intra-patient variations in health status during COPD, and it provides significant variations in the score when distinguishing between the clinical stability and acute clinical exacerbation phases of the disease. It is a coincidence that the correlation with the specific COPD version of the St. George’s Respiratory Questionnaire was particularly high (r = 0.80) [10-12].

It should be underlined that the CAT score proved not affected by the age, gender, body size, and the educational level of COPD patient’s, thus further emphasizing its stability in assessing the patients’ health status.

Furthermore, even if the CAT score obviously proves significantly inversely correlated to the values of lung functions, it is not strictly and uniquely linked to them: in other words, it does not represent, therefore, an alternative measurement to lung function but, on the contrary, it focuses on different areas of the respiratory health in COPD patients, thus providing an useful, objective, and instantaneous tool for the long-term clinical and therapeutic monitoring of COPD patients in the specialist outpatient setting. This is confirmed by the stronger link between the CAT score and the respiratory symptoms (such as dyspnoea and wheezing) reported by the patient.

Unlike previous international investigations, the present study proved a significant relationship between the CAT score and the occurrence of respiratory infections. Together with the significant positive relationship assessed between the CAT score and the frequency of steroid and/or antibiotic courses in the previous year, this feature emphasizes and explains the ability of the CAT questionnaire to highlight the variations in health status due to the occurrence of acute COPD exacerbations.

Finally, the evidence indicating that the CAT score proved to be significantly higher in patients currently untreated, or improperly managed, further suggests that the CAT is a sensitive tool for assessing the COPD patient’s “current” respiratory status.

## Conclusions

The CAT questionnaire is thus confirmed as a simple and reliable tool which is capable of measuring COPD-related health status. Thanks to its unique characteristics, it thus seems particularly useful in the specialist outpatient settings, particularly for long-term clinical and therapeutic monitoring of COPD patients.

Finally, the availability of a reproducible and consistent tool which is able to ensure the quick, easy, and effective patient-to-doctor communication, can only lead to a substantial improvement in the health-related and socio-economic impact of COPD by providing a more appropriate therapeutic management and a more effective governance of COPD.

On the basis of findings from European studies [10-13] (and now also from Italian data), internal consistency tests for the questionnaire confirm that the CAT is a suitable and reliable instrument for measuring patient-assessed COPD-related health status regardless of language differences. This is of particular significance because it ensures the validity and relevance of the CAT as an international cross-cultural assessment tool for comparing the impact of COPD.

## Competing interests

The authors declare that they have no competing interests.
